# Lateral Femoral Neck and Peritrochanteric Fractures: Anatomical Classifications and Pre-Operative Reduction Techniques—A Narrative Review

**DOI:** 10.3390/jfmk11020241

**Published:** 2026-06-17

**Authors:** Giacomo Capece, Gerardo Giudice, Ruggiero Giliberti, Pierluigi Di Cosmo, Giuseppe Pizzi, Luca Lepore, Rosario Junior Sagliocco, Francesco Cuozzo, Emidio Di Gialleonardo, Michele Gison

**Affiliations:** 1 U.O.C. Orthopedics and Traumatology, Ospedale dei Pellegrini, 80134 Naples, Italy; gerardo.giudice@aslnapoli1centro.it (G.G.); ruggilib@hotmail.it (R.G.); pierluigi.dicosmo@aslnapoli1centro.it (P.D.C.); giuseppe.pizzi@aslnapoli1centro.it (G.P.); dott.rosariojuniorsagliocco@gmail.com (R.J.S.); fra.cuoz@gmail.com (F.C.); michele.gison@aslnapoli1centro.it (M.G.); 2 Department of Ageing, Neurosciences, Head-Neck and Orthopaedics Sciences, Orthopaedics and Trauma Surgery Unit, Fondazione Policlinico Universitario Agostino Gemelli, IRCCS, 00168 Rome, Italy; emidiodiggia@gmail.com; 3 Department of Public Health, University of Naples Federico II, 80131 Naples, Italy; luclepore92@gmail.com

**Keywords:** proximal femur fractures, lateral femoral neck fractures, peritrochanteric fractures, AO/OTA classification, Evans–Jensen classification, intradigital fractures, extradigital fractures, fracture fixation, cephalomedullary nail, dynamic hip screw

## Abstract

Lateral femoral neck and peritrochanteric fractures are common and clinically challenging injuries, particularly in the elderly population, with significant implications for morbidity, mortality, and functional recovery. Traditional classification systems are widely used to guide treatment, yet their reproducibility and clinical applicability remain debated. Increasing attention has been directed toward trabecular architecture and its role in fracture behavior and reduction strategies. This review aims to summarize current evidence on classification systems, trabecular-based fracture patterns, pre-operative reduction techniques, and fixation strategies. A narrative review was conducted using PubMed/MEDLINE, Embase, and Scopus databases up to May 2026. Original studies, reviews, and biomechanical investigations focusing on proximal femur fracture classification, reliability, trabecular alignment, reduction techniques, and fixation methods were included. Data were qualitatively analyzed, with emphasis on interobserver reliability, biomechanical implications, and clinical outcomes. Conventional classification systems, including anatomical, Evans–Jensen, and AO/OTA frameworks, demonstrated variable and generally moderate reproducibility, with reported interobserver agreement ranging from approximately κ = 0.30 to 0.60. Emerging evidence highlights the importance of trabecular architecture, distinguishing intradigital fractures—confined within trabecular pathways and relatively stable—from extradigital fractures, which disrupt load-bearing structures and are associated with increased mechanical instability and higher failure rates. Biomechanical and clinical studies indicate that inadequate reduction with trabecular misalignment significantly increases the risk of varus collapse and implant cut-out. Reduction strategies tailored to fracture pattern, such as internal rotation for intradigital fractures and external or combined maneuvers for extradigital patterns, improve alignment and load transfer. In terms of fixation, dynamic hip screws remain effective in stable fractures, whereas cephalomedullary nails demonstrate superior performance in unstable patterns, with lower reoperation rates reported (approximately 5–8% vs. 10–15%). Management of lateral femoral neck and peritrochanteric fractures should extend beyond traditional classification systems to incorporate trabecular biomechanics. Restoration of trabecular alignment, alongside established parameters such as neck–shaft angle and tip–apex distance, is critical for optimizing outcomes. Further prospective studies are needed to validate trabecular-based classifications and standardize reduction strategies.

## 1. Introduction

Fractures of the proximal femur, particularly those involving the lateral aspect of the femoral neck and the peritrochanteric region, remain among the most frequent and clinically challenging orthopedic injuries worldwide [1]. These fractures are a leading cause of morbidity and mortality in older adults and are associated with significant functional decline and prolonged rehabilitation requirements. In the setting of an aging population, their incidence is expected to rise further, imposing an increasing burden on healthcare systems globally [2,3]. From a biomechanical perspective, the proximal femur functions as a complex structural unit, adapting to various combinations of forces transmitted during daily activities and falls. Consequently, the disruption of this unit not only reflects the severity of trauma but also represents a critical determinant of treatment strategy and prognosis [4].

Accurate classification of proximal femur fractures is considered essential for guiding therapeutic decision-making, estimating mechanical stability, and predicting clinical outcomes. Over the past several decades, several classification systems have been developed to describe fracture morphology and to stratify patterns according to presumed stability [5]. The anatomical classification delineates fractures based on the fracture line relative to the capsular insertion, acknowledging the relationship between fracture orientation and vascular supply [6]. The Evans–Jensen system similarly attempts to define fracture stability based on the integrity of the medial calcar and fragment comminution [7], while the widely used AO/OTA classification offers a comprehensive coding framework for femoral head, neck, and trochanteric fractures [8]. Although these systems remain valuable tools for clinical communication and research, their reproducibility in complex fracture patterns continues to represent a recognized challenge. Interobserver and intraobserver agreement studies highlight important limitations of traditional classification schemas. For example, comparisons between the AO/OTA and Evans/Jensen systems have demonstrated only moderate to poor reproducibility, with neither system consistently reaching acceptable reliability thresholds when used by observers of varied experience levels [9]. In one prospective study examining intertrochanteric fractures, both AO/OTA and Evans/Jensen systems failed to achieve reliable agreement on fracture type or stability, underscoring the need for clearer and more consistent classification criteria [10]. These findings suggest that radiographic morphology alone may not fully capture the biomechanical complexity underlying proximal femur fracture patterns.

Beyond conventional classifications, advances in imaging and computational modeling have provided deeper insight into the internal structural determinants of fracture behavior, particularly the role of trabecular architecture. Contrary to the traditional emphasis on the cortical shell as the primary load-bearing element, increasingly sophisticated biomechanical analyses reveal that the trabecular network plays a central role in load transfer and structural integrity of the proximal femur [11]. Finite element models and experimental studies indicate that trabecular bone contributes substantially to the overall strength of the proximal femur, in some analyses accounting for a reduction in mechanical integrity of over 50–60% when trabecular structures are removed [12]. In fact, hip fracture initiation under simulated lateral fall conditions is often observed to begin within the trabecular compartment at the trabecular–cortical interface, rather than within cortical bone alone. These findings underscore the importance of considering the three-dimensional orientation, density, and continuity of trabecular networks when evaluating fracture mechanisms and devising reduction strategies.

In this context, the distinction between “intradigital” and “extradigital” fracture patterns, conceptual frameworks originally described by De Mayo in 1963, gains renewed significance [13]. Rather than describing fracture location solely in terms of its relation to cortical landmarks, “intradigital” fractures are characterized by lines that remain within primary trabecular pathways or “digitations”, maintaining a degree of alignment with native load-bearing trajectories. These patterns tend to preserve some inherent stability and may be more amenable to reduction via internal rotation maneuvers that restore trabecular continuity. “Extradigital” fractures, however, extend beyond these structural trabecular channels, resulting in more pronounced disruption of load pathways and rotational instability; such patterns may benefit from external rotation or combined reduction techniques to realign trabecular systems optimally.

The incorporation of trabecular-based concepts into fracture assessment offers a complementary perspective to conventional radiographic classifications. By considering the relationship between fracture lines and internal load-bearing pathways, surgeons may better understand fracture stability and optimize reduction strategies [14]. This biomechanical perspective may also influence implant selection, whether dynamic hip screw (DHS), cephalomedullary nail, or alternative constructs, according to the specific mechanical environment of the fracture [15].

Against this background, the present narrative review integrates current evidence on proximal femur fracture classification systems, the intradigital/extradigital paradigm, and pre-operative reduction strategies. By combining anatomical, clinical, and biomechanical perspectives, we aim to provide a comprehensive framework that may support surgical decision-making and improve the interpretation of fracture stability and reduction quality.

## 2. Materials and Methods

This study is a structured narrative review designed to provide a comprehensive overview of lateral femoral neck and peritrochanteric fractures, focusing on classification systems, intradigital and extradigital fracture patterns, pre-operative reduction techniques, and fixation strategies.

Although this study is not a systematic review or meta-analysis, a structured and reproducible literature search strategy was applied. Reporting of the search process was informed by PRISMA 2020 guidelines to enhance transparency; however, these guidelines were used solely as a reporting framework and not as a methodological requirement for systematic review conduct [16].

A comprehensive search was performed in PubMed/MEDLINE, Embase, and Scopus databases for studies published between January 2000 and May 2026. The search strategy combined MeSH terms and keywords using Boolean operators to maximize sensitivity, including terms related to proximal femur fractures, classification systems, trabecular alignment, intradigital and extradigital fracture patterns, and reduction and fixation techniques.

Additional relevant studies were identified through manual screening of reference lists from key articles and major reviews.

Given the narrative design of the study, no formal evidence synthesis or quantitative pooling was performed. Data were analyzed thematically to integrate anatomical, biomechanical, and clinical evidence into a coherent conceptual framework.

**Figure 1:**
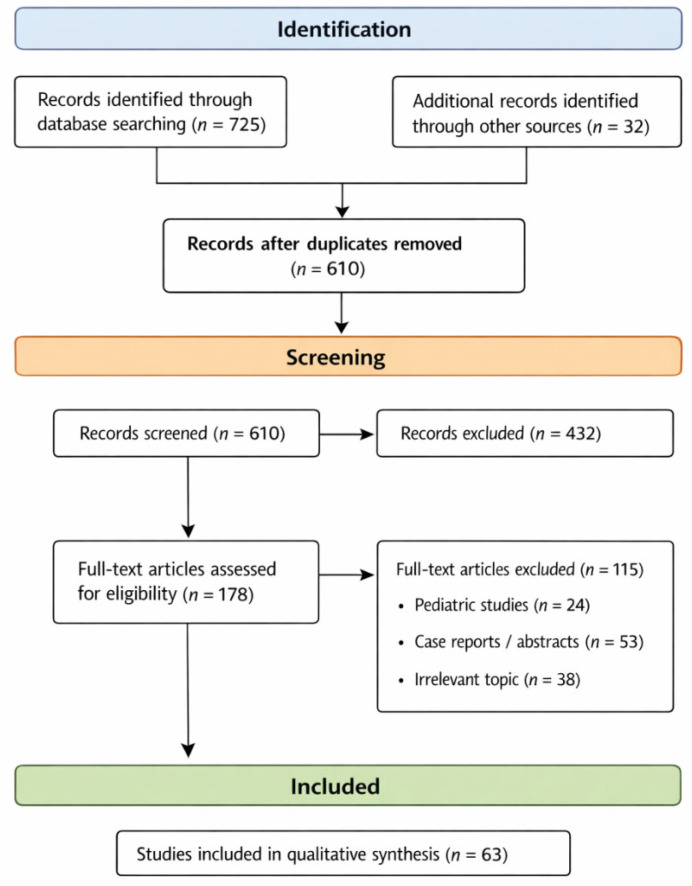
Flow diagram of study selection process (adapted from PRISMA reporting principles).

A study selection flow diagram (Figure 1) is provided to ensure transparency of the screening process; however, it does not imply adherence to a formal systematic review protocol.

### 2.1. Inclusion and Exclusion Criteria

Only articles published in English were considered eligible for inclusion. Eligibility was determined based on whether studies addressed classification reliability, pre-operative reduction techniques, or biomechanical aspects of trabecular alignment in proximal femur fractures. Studies included original research, narrative and systematic reviews, consensus statements, and large multicenter cohort studies. Pediatric fracture studies, case reports, letters, editorials, conference abstracts, and studies not directly related to lateral femoral neck or peritrochanteric fractures were excluded (Table 1).

### 2.2. Search Strategy

All retrieved records were independently screened by two reviewers (G.C. and F.C.). Discrepancies were resolved by consensus with a senior author (M.G.). Extracted data included study design, classification systems, biomechanical findings, reduction techniques, fixation methods, and clinical outcomes. Data synthesis was qualitative and thematic.

## 3. Results

### 3.1. Classification Systems

#### 3.1.1. Anatomical Classification

The anatomical classification stratifies lateral femoral neck and peritrochanteric fractures according to the relationship between the fracture line and the distal capsular insertion [6]. This distinction is clinically relevant, as the vascular supply to the femoral head is closely associated with the retinacular vessels running along the femoral neck. Although lateral fractures are traditionally considered extracapsular, certain patterns, particularly basicervical fractures, may partially involve intracapsular regions, potentially compromising vascularity and influencing healing outcomes, as originally described by Robert S. Garden (1961) [17]. The extracapsular arterial ring surrounding the femoral neck represents a key anatomical structure, and its disruption may correlate with an increased risk of delayed union or fixation failure. Despite its anatomical rationale, this classification lacks standardized validation in terms of reproducibility (Table 2).

#### 3.1.2. Evans and Jensen Classifications

The Evans classification categorizes peritrochanteric fractures based on the integrity of the medial calcar and the degree of comminution, distinguishing between stable and unstable patterns. Jensen later introduced additional subgroups to better capture fracture variability; however, the increased complexity has been associated with reduced reliability [18]. In a study by Pervez et al., both the Jensen and AO classifications demonstrated only fair to moderate agreement, with interobserver kappa values ranging approximately from 0.33 to 0.45 and intraobserver values around 0.48 [19] (Table 2).

#### 3.1.3. AO/OTA Classification

The AO/OTA classification system remains the most widely used framework for proximal femur fractures, dividing them into trochanteric (31-A), femoral neck (31-B), and femoral head (31-C) categories. Its comprehensive structure allows detailed fracture characterization, and several studies have evaluated its reproducibility [8]. Studies evaluating simplified AO/OTA groupings reported interobserver kappa values ranging from approximately 0.40 to 0.60 [20] (Table 2).

### 3.2. Intradigital vs. Extradigital Fractures

The distinction between intradigital and extradigital fracture patterns is based on their spatial relationship to the piriformis fossa and the resulting interaction with the primary trabecular load-bearing system of the proximal femur. Intradigital fractures originate in a more medial and central region relative to the piriformis fossa and are generally confined within the principal trabecular columns. In the studies reviewed, these fractures were described as remaining within the principal trabecular columns and maintaining alignment with the femoral medullary axis.

In contrast, extradigital fractures occur more laterally relative to the piriformis fossa and extend beyond the primary trabecular system. These fractures were described as extending beyond the principal trabecular system and disrupting trabecular continuity.

**Figure 2:**
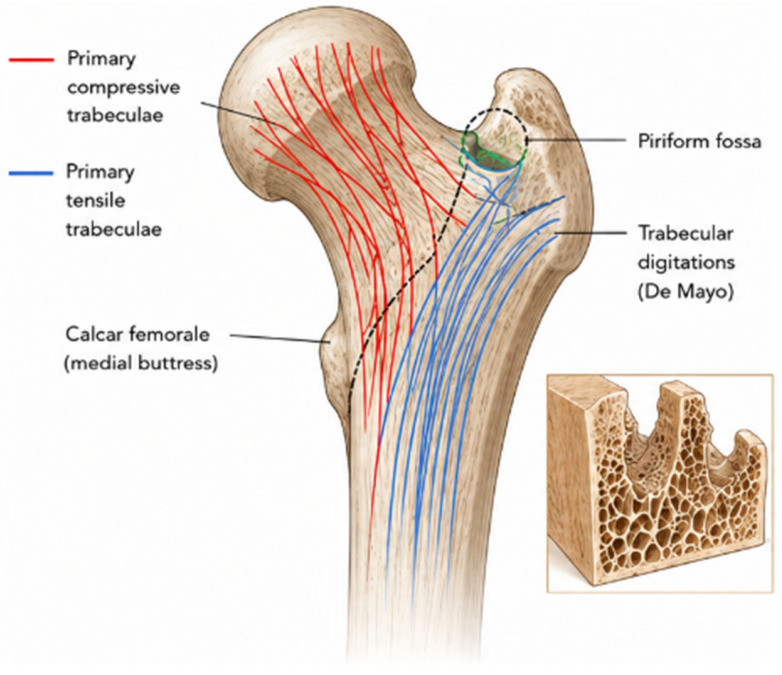
Trabecular architecture of the proximal femur.

**Figure 3:**
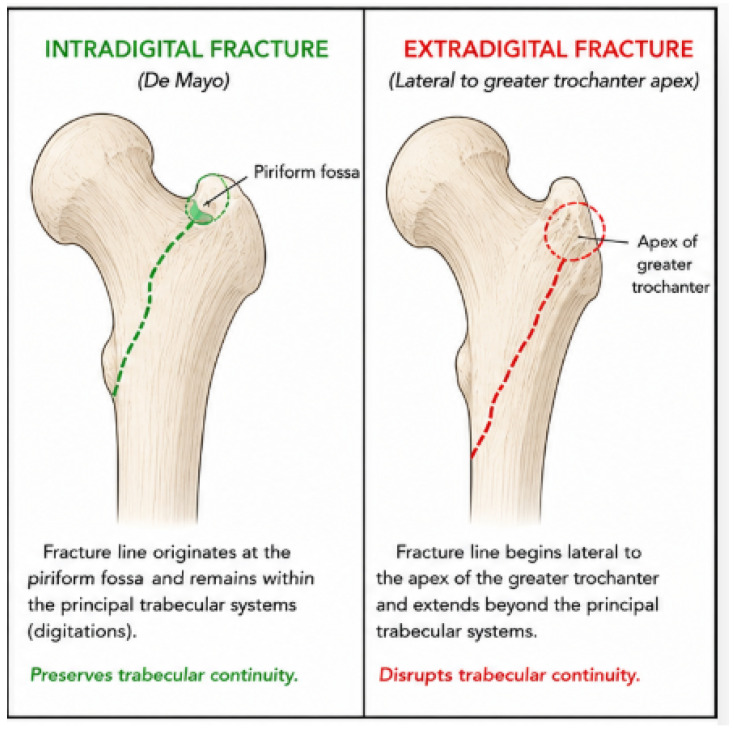
Anatomical basis of intradigital and extradigital fractures.

**Figure 4:**
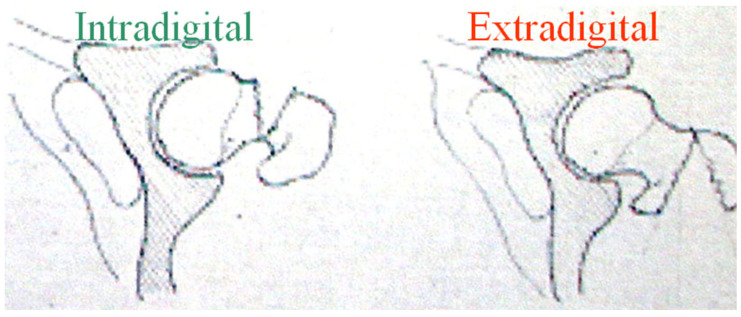
Anatomical basis of intradigital and extradigital fractures in axial views.

Biomechanical studies evaluated the relationship between restoration of trabecular congruency and fixation behavior [21]. Keating et al. reported that inadequate reduction with persistent malalignment is associated with varus collapse and implant cut-out, with increasing failure rates when anatomical or near-anatomical alignment is not achieved [22,23]. The reviewed literature reported different reduction strategies according to fracture configuration, including internal rotation, external rotation, and combined traction–rotation maneuvers (Table 3) (Figure 2, Figure 3 and Figure 4).

### 3.3. Reduction Techniques

Preoperative and intraoperative reduction strategies play a central role in determining clinical outcomes in proximal femoral fracture fixation. Current evidence indicates that restoration of cortical continuity alone is insufficient to ensure mechanical stability. Instead, successful reduction requires re-establishment of the primary trabecular load-bearing system and restoration of the physiological neck–shaft angle, typically ranging between 125° and 135°.

Varus malreduction, particularly when the neck–shaft angle falls below 120°, has been consistently associated with increased rates of fixation failure, implant cut-out, and mechanical overload [24]. Varus malreduction was associated with higher rates of fixation failure and implant cut-out.

In unstable fracture patterns (AO/OTA 31-A2 and 31-A3), studies reported favorable biomechanical parameters when anatomical or slight valgus alignment was achieved.

**Figure 5:**
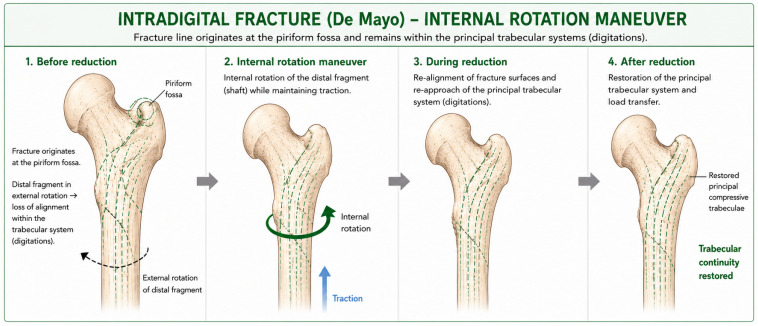
Intradigital fractures, conceptual illustration of reduction maneuvers according to fracture pattern.

**Figure 6:**
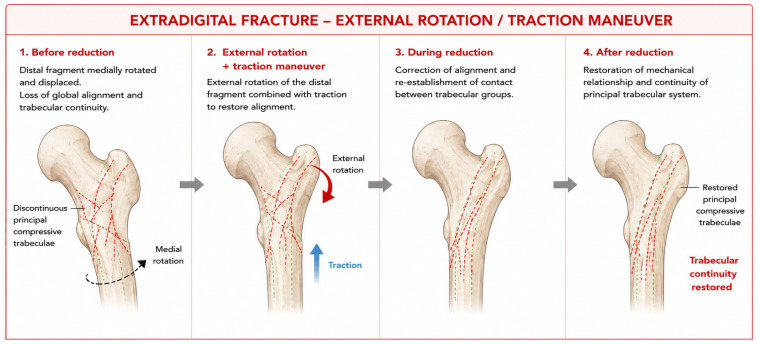
Extradigital fractures, conceptual illustration of reduction maneuvers according to fracture pattern.

From a procedural standpoint, reduction should be interpreted as a vector-controlled maneuver rather than a simple spatial correction. Internal rotation is primarily indicated in intradigital fracture patterns to recenter the distal fragment within the trabecular axis, whereas external rotation and/or combined traction–rotation maneuvers are required in extradigital fractures to restore global alignment and re-establish trabecular continuity (Table 3) (Figure 5 and Figure 6).

### 3.4. Fixation Methods

Dynamic hip screw (DHS) and cephalomedullary nail (CMN) fixation represent the principal surgical options for these fractures. Dynamic hip screw constructs are generally effective in stable fracture patterns but demonstrate higher failure rates in the presence of comminution, reverse obliquity, or lateral wall incompetence. Cephalomedullary nails provide improved biomechanical performance in unstable patterns, including AO 31-A3 fractures and those with subtrochanteric extension, due to their intramedullary load-sharing properties. Comparative studies have shown lower reoperation rates with cephalomedullary fixation in unstable fractures, with values reported around 5–8% compared to 10–15% for dynamic hip screw constructs [15]. A critical technical parameter influencing outcome is the tip–apex distance, first described by Baumgaertner et al., with values below 25 mm associated with a significantly reduced risk of screw cut-out, whereas distances exceeding this threshold correlate with failure rates approaching 20–30% [25,26] (Table 4).

## 4. Discussion

The findings summarized in this review suggest that conventional classification systems remain useful descriptive tools; however, their ability to capture the biomechanical complexity of proximal femur fractures appears limited. The variability observed in interobserver and intraobserver agreement supports the need for complementary approaches that incorporate structural and biomechanical considerations. This narrative review underscores the enduring complexity inherent in the classification and management of lateral femoral neck and peritrochanteric fractures. Despite decades of research and the proliferation of multiple classification schemes, achieving a reproducible and clinically actionable categorization remains elusive [27,28]. The AO/OTA system, while widely adopted in both clinical and research contexts, exhibits limited interobserver reliability, particularly when addressing complex fracture patterns such as multifragmentary trochanteric or basicervical extensions. The moderate intraobserver reproducibility reported in the literature further highlights that even experienced clinicians face challenges in consistently assigning fractures to discrete AO subtypes. These findings suggest that, while the AO/OTA framework provides a comprehensive taxonomy, its practical utility for guiding nuanced surgical decision-making may be constrained by its inherent complexity rather than representing a fully reliable clinical decision tool [9].

The anatomical classification, based on the orientation of fracture lines relative to capsular insertion, retains conceptual value by emphasizing the relationship between fracture morphology and vascular supply. However, its relative simplicity comes at the cost of granularity, particularly in distinguishing subtle variations in fracture instability that influence fixation choice. Similarly, the Evans–Jensen schema, although historically influential for its focus on medial calcar integrity and comminution, demonstrates limitations in capturing the spectrum of fracture morphologies encountered in contemporary practice [29]. Collectively, these observations indicate that traditional classification schemes, while informative, may not fully capture the biomechanical heterogeneity underlying proximal femur fractures and should be interpreted primarily as descriptive rather than mechanistically predictive frameworks.

A key insight emerging from recent literature concerns the role of trabecular architecture in defining fracture behavior and guiding reduction strategies [30]. The intradigital versus extradigital distinction introduces a paradigm shift, moving beyond purely cortical-based classification toward consideration of internal load-bearing pathways. Intradigital fractures, which remain confined within primary trabecular “digitations,” generally preserve rotational stability and demonstrate more predictable responses to internal rotation reduction. In contrast, extradigital fractures disrupt principal trabecular axes, producing rotational instability and a higher risk of comminution. Biomechanical studies indicate that restoration of trabecular continuity is closely associated with improved load transfer and reduced fixation failure, reinforcing the notion that alignment of trabecular systems may be a more sensitive predictor of functional outcome than cortical apposition alone [31]. However, it is important to note that the intradigital/extradigital distinction has not yet been validated in large prospective clinical cohorts, and interobserver reliability remains to be established. Future studies should aim to standardize criteria and assess reproducibility across multiple centers, ideally correlating fracture type with patient outcomes and fixation success.

The analysis of reduction techniques reveals that conventional emphasis on achieving cortical apposition, while necessary, is not sufficient to ensure stability, particularly in complex or extradigital fractures [32]. Successful outcomes appear contingent upon realignment of the principal trabecular systems, often necessitating nuanced application of traction, internal rotation, external rotation, or combined maneuvers. De Mayo’s historical recommendations regarding internal versus external rotation remain foundational, yet contemporary evidence suggests that prospective, quantitative validation of specific reduction protocols aligned with trabecular pathways is still required. Further studies are needed to determine whether advanced imaging techniques can improve preoperative assessment and reduction planning in fractures with variable trabecular involvement [33].

Fixation strategies also reflect the interplay between fracture pattern, trabecular orientation, and mechanical demands. Dynamic Hip Screw (DHS) fixation, while effective for stable A1–A2 fractures and some intradigital patterns, is limited in the presence of severe comminution (AO A3) or subtrochanteric extension involving the trochanteric fossa. Cephalomedullary nails offer mechanical advantages in these scenarios by providing load sharing and improved resistance to rotational forces, highlighting the importance of matching fixation modality to fracture architecture [34]. Metrics such as tip–apex distance and restoration of the neck–shaft angle remain critical determinants of stability, but these must be considered alongside trabecular alignment to optimize outcomes [35,36]. Clinical outcomes should be assessed not only in terms of radiographic union and mechanical complications but also with regard to functional recovery, mobility, pain, and quality of life, particularly in elderly patients. Further clinical studies are required to clarify the role of trabecular-based fracture assessment in surgical planning and to determine whether these biomechanical concepts can improve treatment selection and patient outcomes.

### Study Limitations

This narrative review has several inherent limitations. First, the reliance on published literature introduces potential publication bias, as studies with negative or inconclusive results may be underrepresented. In particular, biomechanical and experimental studies supporting trabecular-based reduction strategies may be preferentially published, potentially overestimating their apparent efficacy and clinical relevance.

Second, the heterogeneity of study designs, including biomechanical analyses, clinical series, and narrative reviews, limits the ability to perform quantitative synthesis or draw firm comparative conclusions. In addition, no formal risk of bias or study quality assessment was performed across the 63 included studies. This methodological limitation should be acknowledged, as it may introduce variability in the strength and reliability of the evidence base.

Third, the qualitative synthesis carries an inherent risk of incorporating overlapping data, particularly when primary studies are also included within secondary narrative reviews, which may lead to partial duplication of evidence.

Fourth, the concept of intradigital versus extradigital fractures, while supported by biomechanical evidence, remains variably defined across studies, and standardized criteria for identification are lacking.

Finally, the review is restricted to English-language publications, potentially excluding relevant data from non-English sources.

Despite these limitations, the synthesis provides a comprehensive and structured overview of current anatomical, biomechanical, and clinical evidence, integrating available data to support a more unified understanding of proximal femoral fracture reduction strategies and to guide future research directions.

## 5. Conclusions

Lateral femoral neck and peritrochanteric fractures remain a complex clinical entity, characterized by variability in morphology, stability, and biomechanical behavior. Traditional classification systems, including the anatomical schema, Evans–Jensen, and AO/OTA frameworks, provide foundational guidance but demonstrate limitations in reproducibility and granularity, particularly for complex or borderline fracture patterns. This review highlights that incorporating trabecular architecture through the intradigital versus extradigital distinction offers a potential biomechanical perspective that may provide additional insights into fracture instability, reduction strategy, and fixation outcomes.

Restoration of the principal trabecular systems, rather than cortical alignment alone, may represent a relevant biomechanical factor influencing mechanical stability and functional recovery. Pre-operative reduction strategies must be interpreted within this biomechanical context, with internal rotation generally indicated for intradigital fractures and external or combined maneuvers for extradigital patterns. Fixation modality should also be selected based on fracture complexity and trabecular integrity, with dynamic hip screws appropriate for stable patterns and cephalomedullary nails favored in comminuted or subtrochanteric extensions. Key operative metrics, including tip–apex distance and neck–shaft angle restoration, remain essential, but must be considered in the context of trabecular alignment to optimize biomechanical conditions and surgical planning.

Advances in imaging technologies, particularly CT-based fracture mapping and three-dimensional reconstructions, offer opportunities to enhance classification precision, identify intradigital versus extradigital patterns pre-operatively, and improve reproducibility in both research and clinical practice. Future prospective studies are warranted to validate reduction protocols tailored to trabecular pathways, assess long-term functional outcomes, and integrate biomechanical modeling with operative decision-making.

A paradigm that integrates anatomical, biomechanical, and clinical insights, including trabecular-based classification, may improve the conceptual understanding and assessment of lateral femoral neck and peritrochanteric fractures, ultimately potentially contributing to more informed surgical decision-making and outcome optimization, while guiding evidence-based surgical practice.

## Figures and Tables

**Table 1:** Inclusion and Exclusion Criteria.

Inclusion Criteria	Exclusion Criteria
Original research articles, narrative and systematic reviews, consensus statements, and multicenter studies.	Case reports, letters, editorials, and conference abstracts.
Studies addressing fracture classification reliability or reduction techniques; biomechanical investigations analyzing trabecular alignment.	Studies not directly related to lateral femoral neck or peritrochanteric fractures.
Publications in English between 2000 and 2026.	Publications not available in English

**Table 2:** Comparison of Classification Systems.

Classification System	Description	Interobserver Reliability (κ)	Intraobserver Reliability (κ)	Key Limitations
**Anatomical**	Based on fracture line relative to capsular insertion and vascular anatomy	Not standardized	Not standardized	Limited validation
**Evans**	Stability based on medial calcar integrity	0.40–0.50	0.50–0.60	Limited detail
**Jensen**	Evans modification with subgroups	0.33–0.45	~0.48	Reduced reproducibility
**AO/OTA**	Comprehensive alphanumeric system (31-A/B/C)	0.30–0.60	0.50–0.65	Complexity, variability

**Table 3:** Intradigital vs. Extradigital Fractures.

Fracture Type	Definition	Trabecular Involvement	Reduction Strategy	Mechanical Behavior	Clinical Implication
**Intradigital**	Confined within trabecular columns	Preserved continuity	Internal rotation	Stable load transfer	Lower failure rate
**Extradigital**	Extends beyond trabecular systems	Disrupted architecture	External/combined rotation	Rotational instability	Higher cut-out risk

**Table 4:** Reduction Techniques and Fixation.

Fracture Pattern	Reduction Technique	Preferred Fixation	Key Parameters	Expected Outcome
**AO 31-A1/A2 (stable)**	Traction + internal rotation	DHS or CMN	TAD < 25 mm; NSA 125–135°	Good stability
**AO 31-A3 (unstable)**	External or combined rotation	CMN	Valgus alignment	Reduced failure
**Extradigital**	Combined rotational maneuvers	CMN	Trabecular realignment	Improved load transfer
**Subtrochanteric extension**	Multiplanar reduction	Long CMN	Control of varus/malrotation	Lower reoperation rate

## Data Availability

All the data we analysed and tables we compiled are available for any clarification.

## Abbreviations

AO/OTA	Arbeitsgemeinschaft für Osteosynthesefragen / Orthopaedic Trauma Association
DHS	Dynamic Hip Screw
CMN	Cephalomedullary Nail
TAD	Tip–Apex Distance
NSA	Neck–Shaft Angle
CT	Computed Tomography
PRISMA	Preferred Reporting Items for Systematic Reviews and Meta-Analyses
